# Recent Methods in the Administration of Anæsthetics

**Published:** 1904-09

**Authors:** Freeman Allen

**Affiliations:** Boston, Mass.


### International Dental Journal.

Vol. XXV. September, 1904. No. 9.

### Original Communications.[1]

### RECENT METHODS IN THE ADMINISTRATION OF ANÆSTHETICS.[2]

BY FREEMAN ALLEN, M.D., BOSTON, MASS.

Mr. President and Gentlemen of The New York Institute of Stomatology,—It is a great pleasure to speak to you upon this subject, because members of your branch of the profession have always been prominently associated, not only with the discovery of anæsthetics, but with promoting the scientific use of them.

You know that Horace Wells, a dentist living in Hartford, Conn., first used nitrous oxide as an anæsthetic in 1844, and that in 1864 Dr. Colton, a New York dentist, continued the use of gas, which had practically ceased with the death of Wells in 1848.

You know also that Dr. Morton, a Boston dentist, first administered ether in 1846 at the Massachusetts General Hospital, and the names of Carlson, a German dentist, and Billeter, a Swiss dentist, are associated with the first considerable use of ethyl chloride as a general anæsthetic in 1897.

---

[1] The editor and publishers are not responsible for the views of authors of papers published in this department, nor for any claim to novelty, or otherwise, that may be made by them. No papers will be received for this department that have appeared in any other journal published in the country.

[2] Read before The New York Institute of Stomatology, May 3, 1904.

Since the discovery of these various agents very many of the improvements in method and apparatus for administering them have come from members of the dental profession.

During the ten years that followed the discovery of gas by Wells, and of ether by Morton, no progress in method and apparatus was made; but in 1858 Dr. John Snow, of England, did some work on the physiological action of chloroform and ether and devised inhalers for their administration.

Still more important work was that of Mr. J. T. Clover, of London, who in 1862 invented a regulating chloroform inhaler, and in 1876 demonstrated the advantage of using gas as a preliminary to ether.

In recent years Dr. Frederick W. Hewitt, of London, has done much to promote the scientific administration of anæsthetics. His most important work, begun in 1886, was the administration of nitrous oxide with definite percentages of oxygen, and in 1894 he perfected an excellent apparatus for this purpose.

So, although Americans discovered gas and ether, yet it appears that since 1858, until very recently, most of the literature of anæsthesia and many of the improvements in methods and apparatus have come from England.

I think the reason for this is the discovery of chloroform by Sir J. Y. Simpson in 1847. Chloroform was found to be a valuable but dangerous anæsthetic, requiring more caution in its administration than gas or ether. The English investigators began by inventing methods and apparatus for the safe administration of chloroform, and, in so doing, their attention was attracted to similar improvements in gas and ether. It became evident to professional men in England that the administration of anæsthetics was too important to be intrusted to inexperienced persons, and men began to devote themselves entirely to this work, with the result that anæsthesia became more systematized than in America.

Since the days of Clover the number of professional anæsthetists in England has greatly increased. To-day in the dental and general hospitals of London anæsthetics are administered almost entirely by professionals. Anæsthesia is systematically taught in the medical schools, and students are required to pass examinations and receive practical training in this line of work before graduation.

Different anæsthetists hold different appointments, just as do surgeons; thus Dr. Frederick W. Hewitt is Anæsthetist to His Majesty, the king; Anæsthetist and Instructor in Anæsthetics at the London Hospital; Anæsthetist at the Charing Cross Hospital and at the London Dental Hospital.

There are societies of anæsthetists who hold regular meetings devoted to the science of anæsthesia; thus Dr. Dudley Buxton is Ex-President of the Society of Anæsthetists; Administrator of Anæsthetics and Lecturer in the University College Hospital; Consulting Anæsthetist to the National Hospital for Paralysis and Epilepsy; Senior Anæsthetist to the Dental Hospital of London.

Many London anæsthetists are men well advanced in years. Dr. Buxton, for instance, is fifty or fifty-five years old, with gray hair and beard, but well preserved and active; and I remember well at the Middlesex Hospital in London a Mr. Norton, who must have been sixty-five or seventy years old, but a careful and skilful anæsthetist, equal to any emergency.

So, although the most useful and important anæsthetics were discovered in America, they were elaborated in England.

In recent years, however, American investigators have taken up this line of work. In 1889 Dr. Thomas L. Bennett, now of New York, but then of Denver, Col., began to investigate the subject of anæsthesia. He used a Clover inhaler for several years, but finally invented his combined gas and ether inhaler, which is far ahead of anything any country has produced for this purpose. He was, I think, the first professional anæsthetist in this country, and has been practising this line of work in New York since 1897. Dr. Bennett has had many imitators, of whom I am one, and interest in anæsthesia has gradually increased; as yet, however, the anæsthetist's profession in this country is not an overcrowded one.

### SECTION II.

The anæsthetics in common use to-day are nitrous oxide, ether, chloroform, mixtures of chloroform and ether, and ethyl chloride. In considering these agents in detail, the most important thing is the question of their safety, which it can do no harm to review.

Of all agents, nitrous oxide, as ordinarily used, is the safest. Considering the innumerable times that it is given, both in this country and in England, exceedingly few deaths are reported. It is considered so safe that most writers make no attempt to estimate its safety in figures. I have seen somewhere its death-rate given as one in two hundred and fifty thousand.

Nitrous oxide and oxygen is a still safer form of anæsthesia, when limited to short operations, but lately anæsthetists, especially in this country, have undertaken to use gas and gas and oxygen for the longer major surgical operations, and have reported administrations of from thirty minutes to two hours and a half.

When thus used the danger of gas, or gas and oxygen, is very materially increased. I have seen very alarming symptoms occur, and, on one occasion, almost lost a patient under this form of anæsthesia. In certain special cases of advanced renal and cardiac disease, however, it gives the patient the best chance, especially if the operation is one which does not require muscular relaxation. My longest case was one of forty-five minutes, the operation being dilating and curetting, ventral suspension, and appendectomy. For long operations, however, I have come to regard this form of anæsthesia as treacherous and as less safe than chloroform.

*Ether.*—Next in safety to gas comes ether. Excepting in people who are distinctly feeble or diseased, ether is perfectly safe. In ether accidents respiration always fails before the circulation, so that, if taken in time and persisted in long enough, artificial respiration will almost always save such cases. The most reliable statistics give the death-rate from ether as one in sixteen thousand.

*Chloroform.*—Chloroform is the most dangerous of all anæsthetics. Besides being a direct cardiac depressant, it has a tendency to cause irregularities in respiration which react upon the heart. These irregularities are seen mostly in the lighter degrees of chloroform anæsthesia, and may be produced by reflex causes. During the stage of excitement the respiration is especially irregular and the patient may suddenly, with a deep inspiration, inhale an overdose; any interruption of respiration during chloroform anæsthesia is therefore dangerous, as the chloroform already in the lungs becomes incarcerated and reacts upon the heart. Reflex causes, such as operative procedures, are apt to produce temporary arrest of breathing and consequent danger, and, as reflexes are more active during a light anæsthesia, a deep chloroform narcosis is, in some ways, safer than a light one; for this reason, too, chloroform is particularly dangerous in mouth, nose, and throat operations, since respiration is very apt to be interfered with in these cases.

Chloroform is especially unsafe when given to a patient who is sitting upright, because, besides being a cardiac depressant, it is a vasodilator, and allows the blood to gravitate from the great centres in the medulla into the dilated splanchnic vessels. Again, although a deep chloroform narcosis is, as I have said, in some ways preferable to a light one, it has the danger of overcoming the heart by an overdose, so chloroform is dangerous from any point of view. Its death-rate is estimated as one in three thousand.

*Mixtures.*—I consider mixtures containing ether and chloroform just as much safer than pure chloroform as they contain more ether than chloroform, provided they are administered with certain precautions which tend to produce an even evaporation of their ingredients; thus Schleich's Mixture No. 3, containing—

Petroleum ether, 15 volumes;
Chloroform, 13 volumes;
Ether, 80 volumes, is comparatively safe if given with ordinary precautions, owing to the preponderance of ether in it. When a house officer at the Massachusetts General I gave this mixture for two months as a routine anæsthetic, having two or three accidents, but no deaths.

The English A. C. E. Mixture, containing—

Alcohol, 1 part;
Chloroform, 2 parts;
Ether, 3 parts, is safer than pure chloroform, though not so safe as Schleich's No. 3.

The best and safest mixture that I know of is the "Anæsthol," recently introduced by Dr. Willy Meyer, of New York. This contains approximately—

Ethyl chloride, 1 part;
Chloroform, 2 parts;
Ether, 3 parts, and is said to be a true molecular mixture, having a boiling-point of 40° C. (104° F.); this secures the almost simultaneous evaporation of the three drugs, and adds materially to the safety of the mixture. Mixtures are not used enough for valuable statistics to be prepared in regard to them. I think they are safer than pure chloroform, although not so safe as ether.

*Ethyl chloride* has lately become popular as an anæsthetic. The most reliable statistics place its death-rate as one in sixteen thousand, but as it has not been used nearly as long as other anæsthetics, statistics in regard to it have not much weight. It is analogous in its action to gas, and my limited experience with it leads me to believe that it is safe if restricted to short operations. It does not produce cyanosis, but seems at times to have a slight depressing effect upon the circulation, especially during the first part of the administration. If we accept its death-rate as one in sixteen thousand, we find it safer than chloroform, as safe as ether, but not so safe as gas.

This gives the following order of safety of the anæsthetics we have been reviewing:

1. Nitrous oxide and oxygen (if limited to short operations).
2. Nitrous oxide.
3. Ether.
4. Ethyl Chloride.
5. Mixtures.
6. Chloroform.
7. Nitrous oxide and oxygen, when used in long operations.

Deaths occurring under anæsthetics should always be reported promptly and in full detail, as this is the surest way of determining the safety of any anæsthetic.

But simply because one agent is statistically safer than another, it should not be arbitrarily used in preference to that other, nor should any one method, such as giving morphine and atropine, be recommended as a routine procedure. As in other branches of medicine, different patients require different agents and methods, and the anæsthetist, having carefully examined his patient, must select and vary his treatment according to age, sex, disposition, habits, condition of the heart, lungs, etc.

To meet the indications of various cases anæsthetics are administered in sequence, as it is called; for example, we use the gas-ether sequence, the ethyl-chloride-ether sequence, the anæsthol-ether sequence, and the gas-ether-chloroform sequence.

Besides the proper selection of the anæsthetic the anæsthetist must concern himself with many details, such as the influence of posture upon the respiration and circulation, the proper protection of the patient during operation against drafts, pressure paralyses, etc., and must attend to the proper stimulation of the patient in case of necessity. He should be held entirely responsible for the safety of the patient as regards the anæsthetic. Ether pneumonia and other sequelæ may also be said to be due to lack of care on the part of the anæsthetist.

### SECTION III.

I will now speak of some practical points in the administration of these agents.

*Gas.*—Gas is so easily eliminated that in order to be effective it must be made to replace the residual air in the lungs, and the access of fresh air must be cut off for the time being. In other words, to produce the best results pure gas and no air must be given, at least for the first part of the administration. The practical difficulty that we encounter in giving gas is not from giving the patient too much gas but from giving too much air, thus delaying anæsthesia. Therefore, in order to be effective, the gas machine must be so made as, first, to keep out air; secondly, to replace residual air in the lungs with gas; and thirdly, to keep a certain amount of gas constantly in the lungs.

The simplest and most effective form of apparatus for this purpose is the one ordinarily seen in the dentist's office where much extracting is done. Gas is liberated from a cylinder and enters a long tube of large caliber which ends in a mouth-piece. This is placed in the patient's mouth and the lips are closed over it. If the nose is also closed the patient inspires pure gas; if the nose is not closed he inspires gas plus a small amount of air; during expiration air plus a certain amount of gas passes through the nose; thus with each inspiration the patient gets more gas; with each expiration he loses more air, so that gas gradually replaces air in the lungs and anæsthesia rapidly results. When the inhalation is stopped there results an anæsthesia of varying duration, which is called the "available" or working anæsthesia. This apparatus is simple and effective, but is not portable, and is suited only for dentistry.

The same result is accomplished in other forms of apparatus which have closely fitting face-pieces to exclude air and inspiratory and expiratory valves; the best form of such an apparatus is Bennett's gas inhaler. Gas is liberated from a cylinder and conducted by a rubber tube to a distensible rubber bag of about two gallons capacity; the closely fitting face-piece is accurately applied, gas is liberated from the bag, and is inspired by the patient through the inspiratory valve; the patient expires through the expiratory valve, the expirations going into the surrounding air and no fresh air entering the lungs during inspiration, the expiratory valve being closed. In this way gas is made with each inspiration gradually to replace the residual air in the lungs. When signs of approaching anæsthesia occur, the gas may be shut off and the valves thrown out of action by turning the stop-cock; the patient breathes back and forth into the bag. The result of this, however, is not asphyxiation, but delaying of asphyxiation, because the patient is breathing a mixture of gas plus what residual air there is left in the lungs, whereas, if he had been permitted to go on breathing gas through valves the gas would have replaced all the air in the lungs and asphyxia would result, so this manœuvre of throwing the valves out of action delays asphyxia and also results in a longer available anæsthesia, because, as a rule, the longer the inhalation the longer the available anæsthesia.

For dentistry, perhaps, this form of apparatus presents little advantage over the simpler form that I at first described. For the longer surgical operations it presents distinct advantages: in the first place, the bag, being near the face-piece, offers no mechanical impediment to respiration; in fact, by keeping the bag well filled, a constant positive pressure is kept up which keeps a steady stream of gas flowing into the lungs without much inspiratory effort on the patient's part. The vents for the admission of air enable us to keep the patient under, and, at the same time, to admit such small definite quantities of air as are necessary to prevent too much cyanosis. In using this machine the most essential point is to select an accurately fitting face-cushion, as even very small amounts of air admitted during the first part of the inhalation will spoil a good gas administration.

It is possible to give gas with suitable proportions of air for long operations, but it usually produces an unsatisfactory form of anæsthesia, owing to the lack of muscular relaxation and the asphyxial symptoms that occur with it. Without discussing the physiological reason for this, the clinical fact is, that it is not possible to give enough air with gas to dispel these asphyxial symptoms and, at the same time, to control the patient. My longest case of gas and air anæsthesia was a nephrectomy lasting thirty-five minutes.

Other methods of giving gas in which you may be interested are (1) Dr. Flux's open method; (2) Dr. Patterson's apparatus for giving gas in long operations on the mouth.

In Flux's method an open cone made of glass or celluloid, with an accurately fitting inflatable rubber-cushion, is used. Gas is allowed to flow into the top of this cone during inspiration only, and, being heavy, sinks at once to the bottom of the cone and is inhaled. In timid persons and children this method is said to produce a tranquil and satisfactory anæsthesia without cyanosis; it consumes a great deal of gas, however, and does not seem to me practical.

Patterson's apparatus I have seen used at the London Dental Hospital. It consists of a gas-cylinder with a foot-key by which gas is liberated through a long rubber tube into a rubber bag of four gallons capacity; at the top of the bag is a stop-cock which turns on gas or air as needed. From the stop-cock two tubes lead to the nose-piece fitted with an inflatable rubber cushion to permit of closer approximation. This is closely applied over the nose and the patient breathes gas. Anæsthesia takes a little longer by this method, owing to the air inhaled through the mouth; if more rapid anæsthesia is desired, there is a separate mouth-piece fitted with an expiratory valve, by which the patient gets no air and the anæsthesia results more rapidly. With the nose-piece only, in about fifteen or twenty seconds; eight or ten gallons are used for an ordinary administration and twenty or thirty gallons last ten or fifteen minutes.

An important advance was made in 1894 when Hewitt perfected his apparatus for the administration of gas with definite percentages of oxygen. You know that nitrous oxide is a pure anæsthetic and is not dependent upon asphyxia for producing its results; this is proved by the fact that by admitting definite percentages of pure oxygen it is possible to keep patients deeply and quietly anæsthetized for long periods of time without any asphyxial symptoms. A suitable patient under this form of anæsthesia appears to be sleeping peacefully, with normal or slightly improved color and tranquil or gently snoring breathing; it gives a longer available anæsthesia in dentistry than does pure gas. (Describes apparatus.)

Ether is commonly administered in one of two methods,—the semi-open method and the closed method. The semi-open method is the one ordinarily seen in hospitals, and consists in giving ether on a cone made of tin or paste-board. The paste-board cones are the best, as they can be accurately fitted to the face. This method is the best for persons who are not experts, but it requires large amounts of ether unless the cone is accurately moulded to the face so as to exclude air.

The principle of air limitation in giving ether was expounded by Clover in 1876 when he invented his inhaler. This is known as the closed method, and in experienced hands is much better than the open method, because it requires very much less ether, and the proportions of air and ether-vapor are under perfect control. The result is that patients are less apt to become "soaked" with ether, and recoveries are much more rapid and free from nausea and vomiting. Bennett's ether inhaler is the best type of the closed inhaler; with it suitable patients can be comfortably and safely anæsthetized with ether in from two to five minutes, and the entire apparatus can be boiled and sterilized without damaging it.

A third, and exceedingly useful, method of giving ether is by the apparatus invented by Dr. Fillebrown, of Boston, or the Harvard Dental School. In dental and oral surgery it is necessary to give ether with the mouth open in order that the surgeon may work while the anæsthesia is being continued. Ether, as ordinarily used, could not control the patient in the presence of so much air; nor is chloroform safe for such cases owing to the danger connected with giving this direct to patients in the sitting posture. Dr. Fillebrown's apparatus heats the ether, thus doubling the rate of its evaporation and intensifying its strength so as to make it effective with large amounts of air. His original apparatus was modified by his assistant, Dr. Rogers, and still further improved by Mr. Lockwood, of Boston. It is exceedingly practical in head cases of all kinds, and will control very difficult subjects. (Demonstration of apparatus.)

Gas and ether, or the gas-ether sequence, is much used in surgery. When properly administered it is a great comfort to both patient and surgeon. Instead of ten or fifteen minutes of struggling, shrieking, coughing, retching, and vomiting the patient may be safely plunged in a profound anæsthesia in two minutes. English statistics estimate the safety of gas and ether as greater than that of ether alone. When gas is used there is much less nausea and vomiting and a more rapid recovery; the patient is spared all the suffocating sensations so often produced by ether alone. This method is useful in dentistry because it is safe in the sitting posture and will give an available anæsthesia which may be anywhere from four to ten times as long as that resulting from gas alone, and a recovery which is often unattended by nausea and vomiting. (Demonstration of Bennett's combined gas and ether inhaler.)

The principles of giving chloroform are exactly the opposite of those for ether. It must, under all circumstances, be administered with large amounts of air, as it is easy to give an overdose. Chloroform is best given on an Esmarch or Schimmelbusch mask, using a drop bottle and the constant drop method. It can also be satisfactorily given on a small, square piece of folded gauze, by the so-called "Scotch" method, which consists in shaking on comparatively large doses of chloroform at intervals. For dental and oral surgery, tracheal chloroforming, etc., many regulating chloroform inhalers have been devised. A good one is Krohner's regulating inhaler. (Demonstration of Krohner's regulating inhaler.)

Mixtures, according to the amount of chloroform they contain, should be given with more or less air; thus the English A. C. E. mixture, having two parts of chloroform and three of ether should be given as chloroform, while Schleich's mixture No. 3 can be given on a cone or other form of semiopen inhaler. As I mentioned before, the danger in mixtures is the different rates of evaporation of their ingredients, but if the mixture has a definite boiling-point and is administered by the drop method, this danger is practically nil.

I have had considerable experience with mixtures, and consider them safer than chloroform.

Much has been written lately about ethyl chloride, which until 1897 was used only as a local anæsthetic. Ethyl chloride is exceedingly volatile, has a low boiling-point,—that is, 12.5° C. (55° F.), —a slight, rather oniony odor, and is inflammable. It is sold in graduated glass vials with patent stoppers under various proprietary names. The commonest kinds are "Antidolorin," "Anæstile," "The Cleveland Gas Company's Agent," "Kelene," and "Narcotile." Of these, I have found that kelene and narcotile give the best results, and I have used narcotile more than any other.

Ethyl chloride is analogous in its action to gas, producing its effects more rapidly and being very rapidly eliminated. It must be given on the same principles as gas,—namely, with almost complete exclusion of air. Ethyl chloride is useful, and I believe safe, in slight operations that do not require muscular relaxation nor a deep anæsthesia. I believe it has been found by American practitioners to be unsatisfactory in dental surgery, on account of the short available anæsthesia that it produces. Dr. Thomas D. Luke, of Edinburgh, however, reports a series of dental cases in which the available anæsthesia varied from one to five minutes, which is longer than that produced by gas. It does not produce cyanosis, but it will not abolish the higher reflexes nor produce muscular relaxation. My longest case with it was one of forty minutes, given for an orthopædic operation on the ankle-joint. This anæsthesia was very ragged, and was followed by intense and persistent nausea and vomiting. My experience with this drug is limited to about sixty cases, which, I regret to say, I did not record systematically, but many of them were followed by nausea and vomiting.

Since many writers, carried away by enthusiasm for ethyl chloride, have claimed that it is superior to gas and will supplant gas, it may be profitable to compare the two. Ethyl chloride, although it seems to be safe, is not so safe as gas, and has not been put to anything like the test for safety that gas has, only having been used since 1897. Ethyl chloride is not so agreeable to inhale as gas; gas has more of a taste than an odor, and patients who have taken both have assured me that they preferred gas. Recoveries from ethyl chloride are much less satisfactory and much more often attended with headache, nausea, vomiting, and a dazed condition of the mind than are recoveries from gas, in which the patient is usually free from nausea and vomiting and perfectly normal at once. So, although ethyl chloride produces no cyanosis, is perhaps less expensive, is more portable, and requires less skill to administer than gas, yet these trifling advantages should have no weight in the face of the three facts I have just mentioned. It may be used to precede ether, but in this respect, also, is inferior to gas. (Demonstration of Ware's and Ash & Sons' inhalers.)

